# Metabolic and morphological assessment of carbon ion radiotherapy response in sacral chordoma

**DOI:** 10.1016/j.phro.2026.101001

**Published:** 2026-05-23

**Authors:** Burte Gantumur, Ayako Taketomi-Takahashi, Sayaka Kodaira, Hiromi Hirasawa, Yasuhiro Fukushima, Kei Shibuya, Yuhei Miyasaka, Tatsuya Ohno, Yoshito Tsushima

**Affiliations:** a Department of Diagnostic Radiology and Nuclear Medicine, Graduate School of Medicine, Gunma University, Maebashi, Japan; b Department of Diagnostic Radiology and Nuclear Medicine, Gunma University Hospital, Maebashi, Japan; c Department of Radiology, Gunma University Hospital, Maebashi, Japan; d Department of Applied Medical Imaging, Graduate School of Medicine, Gunma University, Maebashi, Japan; e Gunma University Heavy Ion Medical Center, Maebashi, Japan

**Keywords:** Sacral chordoma, Carbon-ion radiotherapy, FDG-PET/CT, Treatment response

## Abstract

**Background and purpose:**

Sacral chordoma is a rare and locally aggressive malignant tumor. Carbon ion radiotherapy (CIRT) has emerged as a promising therapeutic modality, but tumor size may not decrease immediately after treatment, presenting challenges in evaluating response. We aimed to evaluate tumor response after CIRT in sacral chordoma patients using quantitative parameters obtained from magnetic resonance imaging (MRI) multi-planar measurements and fluorine-18 fluorodeoxyglucose positron emission tomography/computed tomography (FDG-PET/CT).

**Materials and methods:**

A retrospective analysis was conducted on 35 sacral chordoma patients who underwent CIRT. Baseline follow-up images for up to 60 months post-CIRT were assessed. Dynamic changes in tumor size, long-axis diameter (LAD), short-axis diameter (SAD), and cranio-caudal diameter (CCD) were measured. Additionally, FDG-PET/CT parameters consisting of maximum standardized uptake value (SUV_max_), metabolic tumor volume (MTV), and total lesion glycolysis (TLG) were evaluated.

**Results:**

A minor reduction in mean tumor size was first observed at six months post-CIRT (LAD: 1.2%; SAD: 3.1%; CCD: 3.4%). From 12 to 60 months post-CIRT, inter-measurement differences between LAD, SAD, and CCD were significant (*p* < 0.05), with the largest reduction in SAD (32.8%) and the smallest in LAD (11.0%) at 60 months. In a subgroup of 10 patients, who had FDG-PET/CT, the average monthly reduction was greater for metabolic parameters than for morphologic measurements (SUV_max_: 2.0% monthly; LAD: 0.6% monthly).

**Conclusions:**

Sacral chordomas treated with CIRT exhibited an unequal shrinkage pattern. Among MRI-based plane-specific measurements, SAD demonstrated the greatest reduction. Furthermore, FDG-PET/CT may predict or confirm treatment response before morphological changes become apparent.

## Highlights

• Maximum standardized uptake value (SUVmax) declined faster than tumor diameter.
• Short-axis diameter fell 32.8% vs 11.0% for long-axis diameter at 60 months.
• SUVmax declined 2.0%/month vs 0.6%/month for long-axis diameter.
• SUVmax increased 14.4–34.5% with local failure or severe adverse events.

## Graphical abstract

**Figure:**
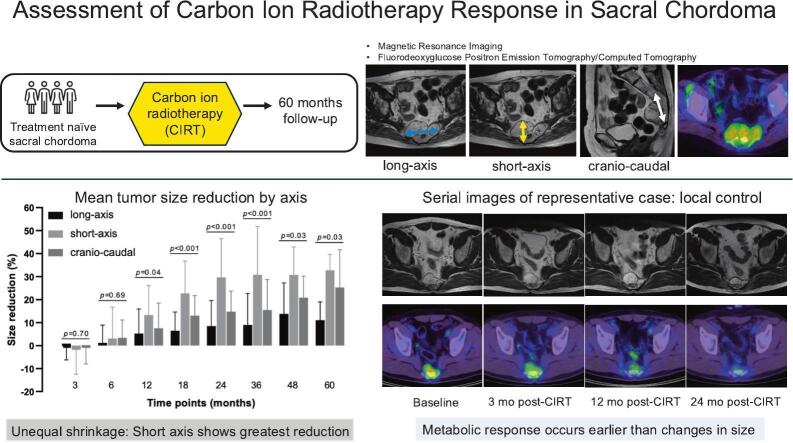

## 1 Introduction

Chordoma is a slow-growing, locally aggressive malignant bone tumor arising from remnants of notochordal cells, with an incidence of approximately 0.8 per million individuals per year [1]. It most commonly occurs in the sacrococcygeal region, accounting for up to 60.7% of all cases [2]. Wide en-bloc surgical resection is the primary definitive treatment for sacral chordoma. However, complete resection is frequently constrained by large tumor size and the anatomical complexity of the sacral region [3], [4]. Carbon ion radiotherapy (CIRT) has emerged as a promising therapeutic modality for chordoma, providing highly conformal and escalated dose delivery to the tumor, facilitated by its high linear energy transfer (LET) and favorable dose distribution conferred by the Bragg peak effect [5]. In recent years, more studies have shown that CIRT has promising effects on inoperable chordoma, with high local control and improved overall survival [6], [7], [8], [9], [10], [11], [12], [13]. Treatment with CIRT has the potential to confer added benefit in patients who undergo incomplete resection or in those eligible for primary photon radiotherapy [14]. Despite clinical benefits, CIRT presents a unique follow-up challenge because the tumor may not be immediately eliminated.

Magnetic resonance imaging (MRI) is the standard for follow-up, valued for its superior soft-tissue contrast and multiplanar capabilities. However, its utility post-CIRT is questionable for several reasons. Tumor regression following radiotherapy is often a slow, gradual process, and local tissue reactions induced by CIRT can further complicate image interpretation. Furthermore, the widely used Response Evaluation Criteria in Solid Tumors (RECIST 1.1) system [15], which is based on measurement changes in a single dimension, i.e., long-axis diameter (LAD), has shown limited accuracy when applied to post-radiotherapy evaluation of sacral chordomas [12], [16]. While volumetric measurement is recognized as an alternative method, it requires additional software and may be more time consuming. Given the limitations of morphologic assessment with MRI, there is a clear clinical need for a more reliable evaluation method.

Fluorine-18 fluorodeoxyglucose positron emission tomography/computed tomography (FDG-PET/CT), which measures metabolic activity with anatomical details, has an increasingly recognized role in the assessment of treatment response following chemoradiotherapy in other tumors [17], [18], [19]. By assessing the metabolic state of the tumor, FDG-PET/CT may be a more accurate indicator of treatment response. However, its role in response assessment of post-CIRT sacral chordoma remains underexplored. This study aimed to evaluate tumor response in sacral chordoma patients after CIRT using multi-planar MRI measurements and quantitative FDG-PET/CT parameters to explore a potential framework for treatment response evaluation.

## 2 Materials and methods

### 2.1 Patient selection

Patients with sacral chordoma without metastases and concurrent malignancy, who received CIRT at our institution following biopsy confirmation from January 2011 to December 2022 were recruited retrospectively. All patients with recurrent disease, history of tumor resection, or lack of baseline or follow-up imaging data were excluded from this cohort. This study was approved by our Institutional Review Board (HS2025-099) and conducted in accordance with the Declaration of Helsinki. Patient consent was waived due to the retrospective study design.

### 2.2 Treatment details

The details of the CIRT procedure used at our institution have been reported previously [6]. The clinical target volume (CTV) included the gross tumor volume (GTV) and subclinical microscopic malignant lesions based on planning computed tomography (CT) with the assistance of enhanced MRI and FDG-PET/CT. The planning target volume (PTV) was defined as the CTV with an additional 5-mm safety margin. All patients received 4.2 Gy relative biological effectiveness (RBE) once a day, four times per week, for a total prescribed dose of 67.2 Gy (RBE) delivered in 16 fractions. The clinical radiation dose was determined using the mixed-beam-model, in which the physical dose is multiplied by the RBE of carbon-ion beams (RBE = 3 at a reference LET), and is reported in units of Gy (RBE)^1^ [20]. A surgical spacer was placed in two patients prior to CIRT to prevent radiation-induced intestinal injury.

### 2.3 Tumor response assessment

All patients (n = 35) had baseline and at least one follow-up MRI after treatment, with follow-up MRIs performed up to 60 months after treatment. The follow-up scans were performed every 3–6 months within the first two years and then every 12 months. To characterize the tumor response by MRI, sizes in three dimensions were used, which were: 1) LAD, which was the tumor’s longest diameter measured on axial images, 2) short-axis diameter (SAD), which was perpendicular to the LAD, and 3) cranio-caudal diameter (CCD), the tumor’s longest diameter along the body’s horizontal plane measured on sagittal images. Changes in size were expressed separately as percent change from baseline. The LAD was used to categorize responses according to RECIST 1.1. According to these criteria, lesions presenting a reduction of at least 30% from the baseline were considered to have a partial response (PR); lesions presenting an increase of 20% from the baseline were considered progressive disease (PD); lesions between those two limits were stable disease (SD).

From the study population, 10 patients who had both a baseline and at least one post-treatment FDG-PET/CT scan acquired at our institute were subgrouped. Follow-up imaging was performed at three months (n = 9, 90%), 7–12 months (n = 7, 70%), and 19–24 months (n = 3, 30%) after CIRT. For these patients, maximum standardized uptake value (SUV_max_), metabolic tumor volume (MTV), and total lesion glycolysis (TLG) were evaluated using a semi-automatic software, Syngo.via (Siemens Healthineers, Erlangen, Germany). To quantify MTV, patient-specific absolute SUV thresholds fixed from the baseline images were used as a reference. The baseline threshold was set at 41% of SUV_max_ according to the European Association of Nuclear Medicine (EANM) guidelines [21]. Calculation of TLG was performed automatically using Syngo.via software as the product of MTV and SUV_mean_ within the MTV (TLG = MTV × SUV_mean_). Changes of these parameters were expressed as percent change from baseline.

Multi-planar measurements of MRI and FDG-PET/CT parameters were performed by a radiologist with 5 years of experience under the supervision of a radiologist specializing in nuclear medicine with 11 years of experience.

### 2.4 Survival and outcome

Local control (LC), overall survival (OS), and progression-free survival (PFS) over a period of 5 years were considered. The OS was the time from the end of treatment to death from any cause. Local recurrence (LR) was defined as enlargement of the tumor or any new separate lesion(s) within the clinical target area. When the core of a new lesion was within the 50% isodose line, the lesion was considered to be LR, but when it was outside the CTV, it was considered regional recurrence. Progression of disease consisted of LR and regional/distant failure. If disease progressed, further follow-up examinations after progression were not considered. The patients lost during follow-up were included up to the time of their last follow-up. Post-treatment toxicities were evaluated using the Common Terminology Criteria for Adverse Events version 4.0 (CTCAE v.4).

### 2.5 Statistical analysis

Kaplan-Meier survival curves were used to show rates of LC, PFS and OS. One-way ANOVA test was used to compare reduction differences between tumor size at different time points. Mixed linear regression analysis was used to model changes of tumor size and metabolic activity over time. Statistical analyses were performed using JMP® Pro17 (SAS Institute Inc., Cary, North Carolina, USA) and GraphPad Prism version 10 (GraphPad software, Boston, Massachusetts, USA) and *p*-values less than 0.05 were considered statistically significant.

## 3 Results

The characteristics of all 35 patients are summarized in Table 1. The median follow-up was 50 (range, 7–118) months. The 5-year OS, PFS, and LC rates were 84.0%, 40.9% and 72.6%, respectively (Fig. S1). The median time for LR was 27 months. CTCAE v.4 grade 3 and above adverse events were detected in three patients (8.6%).

**Table 1:** Patient characteristics.

Characteristic	Total number of patients n = 35
Age (y), median (range)	70 (43–84)
Sex, n (%)	
Male	24 (69)
Female	11 (31)
CIRT dose/fractionation (GyE/fr), n (%)	
67.2/16	35 (100)
Spacer placement, n (%)	2 (5.7)
Pre-treatment tumor characteristics, mean (range)	
GTV (cm^3^)	369.0 (46.2–2074.3)
CTV (cm^3^)	626.8 (164.5–3416.0)
LAD (cm)	8.8 (4.9–20.0)
SAD (cm)	6.5 (1.7–11.8)
CCD (cm)	7.4 (4.6–13.1)
SUVmax	4.1 (2.6–5.9)
MTV (cm^3^)	211.8 (57.7–538.1)
TLG (SUV × cm^3^)	497.3 (158.7–1451.6)

Abbreviations: n (number), y (year), CIRT (carbon ion radiotherapy), GyE/fr (gray equivalent per fraction), GTV (gross tumor volume), CTV (clinical target volume), LAD (long-axis diameter), SAD (short-axis diameter), CCD (cranio-caudal diameter), SUV_max_ (maximum standardized uptake value), MTV (metabolic tumor volume), TLG (total lesion glycolysis).

A total of 198 MRI and 29 FDG-PET/CT examinations were reviewed in this study. The number of MRI evaluations per patient ranged from two to nine, with a median of six. According to RECIST 1.1, among the patients without LR during the 60-month follow-up period (n = 28), there was only one patient (3.6%) who achieved PR (32.0% reduction at the 35th month after CIRT) and the rest were categorized as SD (96.4%). Among patients with LR (n = 7), five were categorized as PD (71.4%), and two were categorized as SD (28.6%) when using RECIST 1.1.

**Fig. 1:**
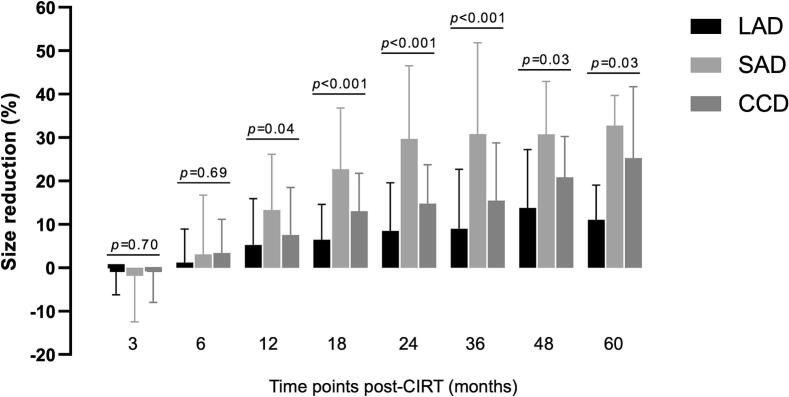
Comparison of mean tumor size reductions in three orthogonal axis measurements at different time points. Differences were analyzed using one way ANOVA. Abbreviations: LAD (long-axis diameter), SAD (short-axis diameter), CCD (cranio-caudal diameter), CIRT (carbon ion radiotherapy).

The mean percentage reductions in tumor size, calculated from three orthogonal axis measurements (LAD, SAD, and CCD) at different time points after CIRT are shown in Fig. 1. Minor increases in mean tumor size were noted at the end of the third month across all three parameters (LAD 0.2%; SAD 1.9%; CCD 0.8%). From six months post-CIRT, minor shrinkage (LAD 1.2%; SAD 3.1%; CCD 3.4%) started to occur. By the end of month 60, SAD showed the greatest reduction with a mean decrease of 32.8%, followed by CCD at 25.2%, while LAD had only shrunk by 11.0%. Inter-measurement differences of these three dimensions showed significance from 12 to 60 months post-CIRT (*p* < 0.05).

**Fig. 2:**
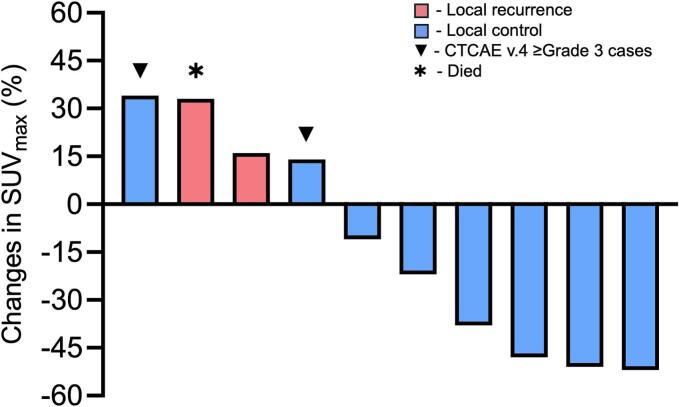
Relative percent changes in SUV_max_ and corresponding 5-year outcomes in the FDG-PET/ CT available subgroup. Each block represents an individual patient, and block height indicates the relative percent change in SUV_max_ from baseline. Red and blue bars indicate local recurrence, and local control, respectively. Abbreviations: CTCAE v.4 (common terminology criteria for adverse events volume 4.0). (For interpretation of the references to colour in this figure legend, the reader is referred to the web version of this article.)

In the FDG-PET/CT available subgroup (n = 10), all patients were categorized as SD according to RECIST 1.1 at 24 months post-CIRT. There were six patients with decreased SUV_max_ (range 10.9–51.9%) and four patients with increased SUV_max_ (range, 14.4–34.5%) as of their last follow-up studies (Fig. 2). All cases with decreased uptake were progression free, with no high-grade post-radiotherapy adverse events, and all patients censored alive at 5-years post treatment (Fig. 3.A, representative case). As for cases with increased uptake, two had LR, and one of these two patients died due to disease progression. The third patient had grade 3 adverse reactions (including post-irradiation sacral infection and rectal perforation), at 17 months post CIRT, which required surgical intervention. The last patient with increased SUV_max_ also developed a deep cutaneous abscess and necrosis of the skin within the irradiated field during the 5-year follow-up period, and subsequently died at 63 months post-CIRT (grade 5 in CTCAE v.4). Each patient’s metabolic and dimensional change over a 24-month period are shown on spaghetti plots (Fig. 4) and their mixed linear regression analysis results are shown on Table 2. The LAD, SAD and CCD decreased by 0.6% (95% CI: 0.4–0.8), 1.5% (95% CI: 1.2–1.7) and 0.9% (95% CI: 0.7–1.1) per month, respectively. In contrast, SUV_max_, MTV and TLG decreased by 2.0% (95% CI: 1.2–2.8), 5.1% (95% CI: 3.1–7.2) and 5.1% (95% CI: 3.1–7.2) by per month, respectively.

**Fig. 3A:**
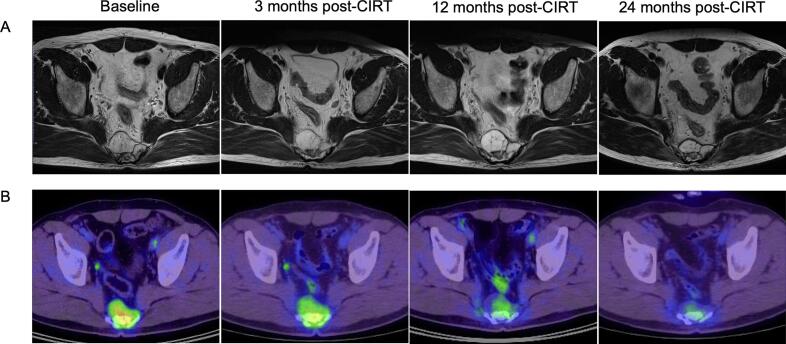
Representative case of tumor response in a patient with local control. MRI and FDG-PET/CT images of a 55-year-old male with sacral chordoma treated with CIRT. A: MRI-T2WI, axial plane. The LAD of the tumor decreased by 0%, 0% and 6% at 3, 12 and 24 months post-CIRT, respectively. B: FDG-PET/CT-axial plane. SUV_max_ decreased by 25%, 45% and 62% at 3, 12 and 24 months post-CIRT, respectively.

**Fig. 3B:**
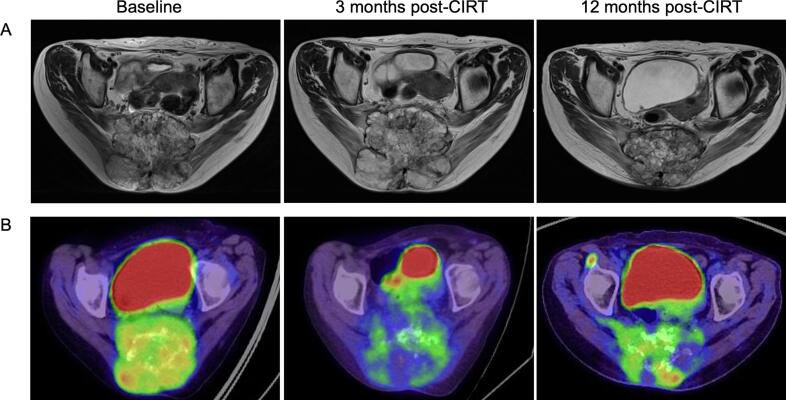
Representative case of tumor response in a patient with local failure. MRI and FDG-PET/CT images of a 72-year-old female with sacral chordoma treated with CIRT. A: MRI-T2WI, axial plane. The tumor’s LAD changed by +9% and −7% at 3 and 12 months post-CIRT, respectively. B: FDG-PET/CT-axial plane. The tumor’s SUV_max_ changed by −35% and +33% at 3 and 12 months post-CIRT, respectively.

**Fig. 4:**
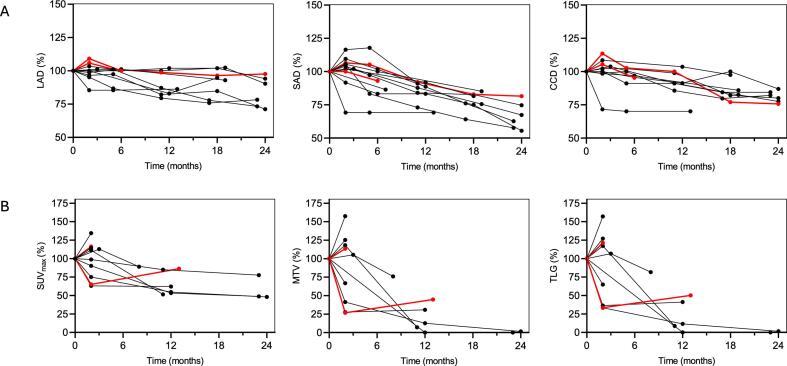
Tumor response curves of subgroup (n = 10) based on dimensional analysis (A) and metabolic analysis (B). Each line represents a single patient. Red lines represent patient with local failure. Abbreviations: LAD (long-axis diameter), SAD (short-axis diameter), CCD (cranio-caudal diameter), SUV_max_ (maximum standardized uptake value), MTV (metabolic tumor volume), TLG (total lesion glycolysis).

**Table 2:** Mixed linear regression analysis.

	Slope (/month)	95% CI	*p*-value
LAD	−0.6	(−0.8, −0.4)	<0.001
SAD	−1.5	(−1.7, −1.2)	<0.001
CCD	−0.9	(−1.1, −0.7)	<0.001
SUVmax	−2.0	(−2.8, −1.2)	<0.001
MTV	−5.1	(−7.2, −3.1)	<0.001
TLG	−5.1	(−7.2, −3.1)	<0.001

Abbreviations: LAD (long-axis diameter), SAD (short-axis diameter), CCD (cranio-caudal diameter), SUV_max_ (maximum standardized uptake value), MTV (metabolic tumor volume), TLG (total lesion glycolysis).

## 4 Discussion

In this study, dynamic changes in the size and metabolic activity of sacral chordoma were evaluated. Significant inter-measurement differences were observed among the three orthogonal axes, with the greatest reduction seen in the short-axis diameter, while the long-axis diameter showed the least response. In the subgroup of 10 patients who underwent FDG-PET/CT, average monthly reduction was greater for metabolic activity than morphological size.

Clinical outcomes demonstrating high local control and low toxicity rates for sacral chordoma treated with CIRT have been consistently reported by heavy-ion beam centers worldwide. Our survival analyses may further support the therapeutic efficacy of CIRT. However, if RECIST 1.1 is used for evaluation, only one patient in our cohort would have achieved a partial response, while the majority would have remained stable over a 5-year follow-up period, despite its high local control. This aligns with prior reports [12], [16], suggesting that LAD has limited utility in assessing radiotherapy response in sacral chordoma. Based on post-treatment biopsy assessments, Evangelisti et al. [22] proposed that tumor size stability should be considered a marker of treatment success. However, they mentioned that their study did not perform biopsies based on PET-positive hotspots and may have missed viable tumor cells. Also, in our cohort, tumor size slightly decreased, but disease progression was detected by increased metabolic activity on FDG-PET/CT (Fig. 3.B) or cases in which tumor regrowth occurred after a period of stability. While post-radiotherapy biopsy could clarify treatment response in stable disease, it is invasive and carries a high risk for seeding in chordoma [23], [24]. In order to explore a potential framework for treatment response evaluation, we analyzed two more orthogonal dimensions which are used routinely together with the longest diameter.

In our study, all LADs were measured in the mediolateral axis, corresponding to the width of the sacrum. All SADs were measured in the anteroposterior axis, which included exophytic soft tissue compartments on axial images. Notably, SAD demonstrated a greater degree of response compared with LAD. This can be partially explained by the findings of Kabolizadeh et al. [12] in which tumor regression is more pronounced in soft tissue compartments than in bone-involved areas after radiotherapy. In our cohort, six months after radiotherapy, slight but uniform shrinkage occurred in all directions. However, over time, LAD reduction was constantly the lowest (Fig. 1). This limited reduction may be attributable to secondary destructive changes involving the sacral bone, while regression of the exophytic soft tissue compartment resulted in progressive shortening of the SAD. Destructive changes in the affected bone, both from the osteolytic activity of the tumor itself and from radiation-induced structural alterations, may persist as signal abnormalities on MRI—even after effective tumor cell kill—potentially overestimating residual tumor size.

In the subgroup of 10 patients with available FDG-PET/CT data, only three patients underwent follow-up FDG-PET/CT at 24 months after CIRT, as FDG-PET/CT is not currently used on a routine basis. However, among the patients who were imaged, an earlier and stronger reduction in metabolic activity was observed compared to changes in MRI parameters. To our knowledge, this is the first study evaluating dynamic metabolic change in post-CIRT sacral chordoma. Evangelisti et al. [22] reported that differences between baseline and 6–12 months post-treatment SUV_max_ and MTV were statistically different. This study could provide clinicians with expectations of monthly relative changes in dimension and metabolic activity of the tumor post-treatment. SUV_max_ decreased approximately three times faster per month than LAD. Not only SUV_max_, but also MTV and TLG were markedly decreased monthly. For patients in whom SUV_max_ increased, outcome was unfavorable in all cases. Mechanistically, FDG is a glucose analog, whose uptake reflects both tumor glycolytic activity and inflammatory cell metabolism [25], [26]. High inflammatory responses after radiation therapy may result from immune system overactivation, and potentially lead to toxicity through several molecular and cellular pathways [27]. This dual sensitivity offers a logical explanation for its overlap with recurrence and post-radiotherapy high-grade adverse events.

The main limitation of this study was the small number of patients with available FDG-PET/CT data and the few cases of LR, and as a result, statistical analysis was limited. This is largely attributable to the rarity of chordomas and the fact that FDG-PET/CT is not routinely used for follow-up. Measurements from FDG-PET/CT in chordomas are moderately heterogeneous, with a lower baseline SUV than in many other tumors. Test–retest analyses indicate that SUV changes must exceed 25–33% to be confident they reflect true biological response [28]. The lower signal-to-noise ratio in chordomas suggests that sample sizes comparable to or larger than those used in breast cancer PET response studies (50–100 patients) are needed for statistically robust evaluation of early metabolic versus morphological changes. Expanding the use of FDG-PET/CT and conducting studies in larger cohorts with longer follow-up periods may be essential to further validate and strengthen its role. Also, this study used the mixed-beam-model to calculate the RBE-weighted dose, which is the standard model implemented in our treatment planning system. Nevertheless, RBE estimation is model-dependent, and alternative approaches, such as the Local Effect Model (LEM), may result in RBE-weighted dose distributions of different effectiveness [29]. Therefore, comparison with studies using other RBE models should be performed with great caution.

In conclusion, sacral chordomas treated with CIRT exhibited an uneven pattern of shrinkage. The anteroposterior axis (SAD) was a more reliable measurement than the mediolateral axis, which corresponds to the tumor’s longest diameter. In addition, our results suggest that FDG-PET/CT may have potential to predict or confirm treatment response before morphological changes become apparent. While these findings are not sufficient to support any changes in clinical practice, combining multi-planar MRI measurements with functional imaging markers, such as SUV_max_, during follow-up could provide a more practical and clinically informative approach to evaluating treatment response. These findings support the need for multicenter, prospectively powered studies to clarify the comparative value of FDG-PET/CT in treatment response assessment and justify any changes in current clinical practice.

## 5 Declaration of generative AI and AI-assisted technologies in the manuscript preparation process

During the preparation of this work the authors used ChatGPT for English language editing. After using this tool/service, the authors reviewed and edited the content as needed and take full responsibility for the content of the published article.

## 6 Submission declaration and verification

We declare that this manuscript is an original work and has not been published previously, in whole or in part, in any form. It is not under consideration for publication elsewhere. All authors have read and approved the final version of the manuscript and agree with its submission to Physics and Imaging in Radiation Oncology.

## CRediT authorship contribution statement

**Burte Gantumur:** Writing – original draft, Investigation, Formal analysis, Data curation. **Ayako Taketomi-Takahashi:** Writing – review & editing, Validation, Supervision. **Sayaka Kodaira:** Investigation. **Hiromi Hirasawa:** Writing – review & editing, Validation, Supervision, Methodology, Conceptualization. **Yasuhiro Fukushima:** Visualization, Data curation. **Kei Shibuya:** Writing – review & editing. **Yuhei Miyasaka:** Investigation. **Tatsuya Ohno:** Resources, Methodology. **Yoshito Tsushima:** Writing – review & editing, Validation, Supervision, Project administration, Conceptualization.

## Declaration of competing interest

The authors declare that they have no known competing financial interests or personal relationships that could have appeared to influence the work reported in this paper.

## Footnotes

1 Relative biological effectiveness (RBE)-weighted doses in carbon ion radiotherapy were expressed in Gy (RBE), consistent with the International Commission on Radiation Units and Measurements (ICRU) Report 93 definition.
