# Supplementary material for: Metabolic and morphological assessment of carbon ion radiotherapy response in sacral chordoma
Source: Phys Imaging Radiat Oncol. 2026 May 23;39:101001. doi: 10.1016/j.phro.2026.101001 (PMC13235522; doi:10.1016/j.phro.2026.101001)

Supplementary material

Figure S1. Overall survival (OS), local control (LC), progression-free survival (PFS) based on the Kaplan-Meier method.

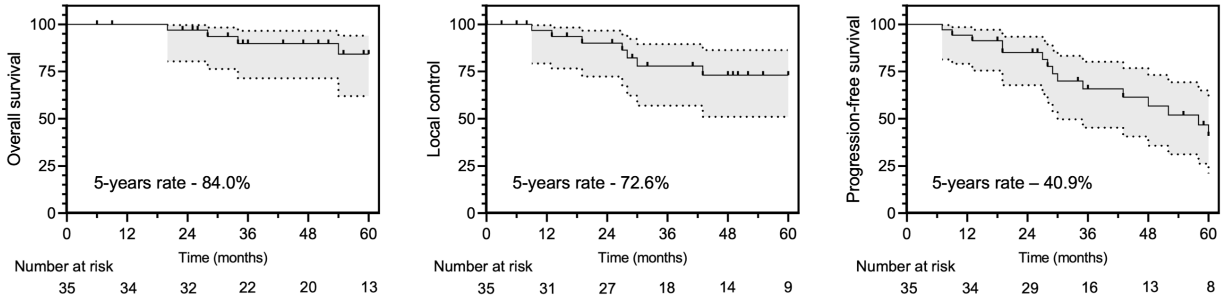

Supplement: Supplementary data 1 [file mmc1.pdf]
